# *Rhodotorula mucilaginosa* JAASSRY1 Ameliorates Cyclophosphamide-Induced Immunosuppression by Regulating Gut Microbiota and Activation of Spleen TLR4/MyD88/NF-κB Pathway

**DOI:** 10.4014/jmb.2510.10031

**Published:** 2026-01-21

**Authors:** Min Yang, Yuguang He, Xinyu Miao, Mubai Sun, Honghong Niu, Mei Hua, Da Li, Hongyan Xu, Jinghui Wang

**Affiliations:** 1 Agronomy of Food Science and Technology, Yanbian University, Yanji 133002, Jilin, P.R. China; 2 Institute of Agro-product Process, Jilin Academy of Agricultural Science (Northeast Agricultural Research Center of China), Changchun 130033, Jilin, P.R. China

**Keywords:** *Rhodotorula mucilaginous*, Immunosuppression, Gut microbiota, Spleen, TLR4/MyD88/NF-κB pathway, Bcl-2/Bax pathway

## Abstract

The present study was designed to evaluate the ameliorative effects of *Rhodotorula mucilaginosa* JAASSRY1 (JAASSRY1) on cyclophosphamide (CTX)-induced immunosuppression in mice. Immunocompromised mice were established by intraperitoneal injection of CTX (80 mg/kg/bw) for three consecutive days, followed by JAASSRY1 orally administered of JAASSRY1 for 21 days. Various immunological parameters, including immune organ indices, spleen cytokine levels, and immunoglobulin profiles, were evaluated. JAASSRY1 prevented CTX-induced immune damage by reversing weight loss and immune organ atrophy, suppressing the expression of IL-6, IL-17, and IFN-γ in the spleen (*P*< 0.01), and restoring levels of IgA and IgG, while up-regulating IL-4 (*P*< 0.01). Furthermore, JAASSRY1 attenuated immunosuppressive spleen injury by modulating the TLR4/MyD88/NF-κB pathway and regulating the Bax/Bcl-2 ratio. JAASSRY1 also alleviated CTX-induced dysbiosis by enhancing the abundance of *Colidextribacter* and reducing the levels of *Parabacteroides* and *Bacteroides*. A significant association was observed between specific gut microbiome *Bacteroides* and immune parameters (*P*< 0.01). Above all, JAASSRY1 demonstrates efficacy in ameliorating immunosuppression through the modulation of the “gut microbiota-spleen” axis, providing a basis for the development of probiotic formulations with immunomodulatory properties.

## Introduction

Antibiotics are widely employed in animal husbandry for disease prevention, treatment, and enhancing production efficiency. However, prolonged and excessive use fosters drug-resistant bacteria, which can transfer to humans and diminish the efficacy of antibiotics for treating human diseases [1, 2]. Some of these bacteria are zoonotic, posing significant risks to human health [3]. To combat this pressing issue, numerous countries and regions, including the European Union, the United States, South Korea, and China, have implemented bans or restrictions on antibiotic use in livestock [4]. Consequently, there is an urgent need to identify effective alternatives, particularly safe and efficient probiotic preparations, for use in livestock and poultry production.

The immune system is essential for animal defense, with the spleen being a central component in vertebrates. As the largest peripheral lymphoid organ, the spleen is crucial for immune regulation, hematopoiesis, and inflammatory responses, thus playing a significant role in systemic immunity. The intestine, as the largest immune organ, relies on the gut microbiota to maintain its immune functions [5]. Disruption of gut microbiota can lead to reduced immunoglobulin secretion, compromised intestinal barriers, bacterial translocation, and various immune-related diseases [6]. Recently, the interplay between gut microbiota and the immune system has garnered significant attention. Additionally, the microbiota influences spleen development and structure [7]. It mediates spleen immune function by modulating chemokine expression and the proliferation and differentiation of B and T lymphocytes [8, 9].

*R. mucilaginosa*, a species within the *Rhodotorula* genus, is prevalent in animals, plants, lakes, and marine environments. Its cells are abundant in nutrients and bioactive compounds, including proteins, natural carotenoids, polyunsaturated fatty acids, and vitamins, providing a vital nutritional source for animals [10]. As a high-quality feed additive, *R. mucilaginosa* enhances animal production, boosts immune function, and increases antioxidant capacity, offering significant market potential and economic benefits [11, 12]. It can substitute for carotenoids, improve egg quality, and maintain chicken gut microbiota balance, with β-glucan metabolites likely contributing to these improvements [13]. Yeast cell wall polysaccharides, known for their prebiotic and nutritional properties, can activate the host immune system [14, 15]. *R. mucilaginosa* ZTHY2 notably enhances immune function and regulates gut microbiota in mice [12]. Research by Hu Ping *et al*. indicates that *R. mucilaginosa* improves growth performance, antioxidant capacity, gastrointestinal function, and gut microbial balance in piglets [16]. Additionally, its carotenoids aid pigment deposition and bolster immunity [17]. Despite these achievements, further research is required to explore whether *R. mucilaginosa* can alleviate immunosuppression-related symptoms via the "gut microbiota-spleen" axis.

This study developed a CTX-induced immunocompromised mouse model to evaluate the effects of *R. mucilaginosa* on the spleen and intestine using multi-omics integration. The preliminary exploration of its protective mechanisms offers a theoretical foundation for its potential use as a probiotic.

## Materials and Methods

### Preparation of *Rhodotorula mucilaginosa* Suspension

The *Rhodotorula mucilaginosa* JAASSRY1 isolated from oil-contaminated soil in Northeast China and collected in China General Microbiological Culture Collection Center (CGMCC; Accession No: 22900). The strain was cultured in modified LB liquid medium at 30°C and 180 rpm for 60 h. The resulting culture was then centrifuged at 4,000 rpm for 10 min to remove the supernatant. The bacterial cells were subsequently washed three times with sterile saline, followed by centrifugation and discarding of the supernatant after each other. The bacterial cells were washed three times with sterile saline and centrifuged again. Finally, the cells were resuspended in sterile saline, adjusting concentrations to 1.0 × 10^6^ CFU/ml and 1.0 × 10^10^ CFU/ml for subsequent use.

### Establishment and Grouping of Animal Models

**Fig. 1:**
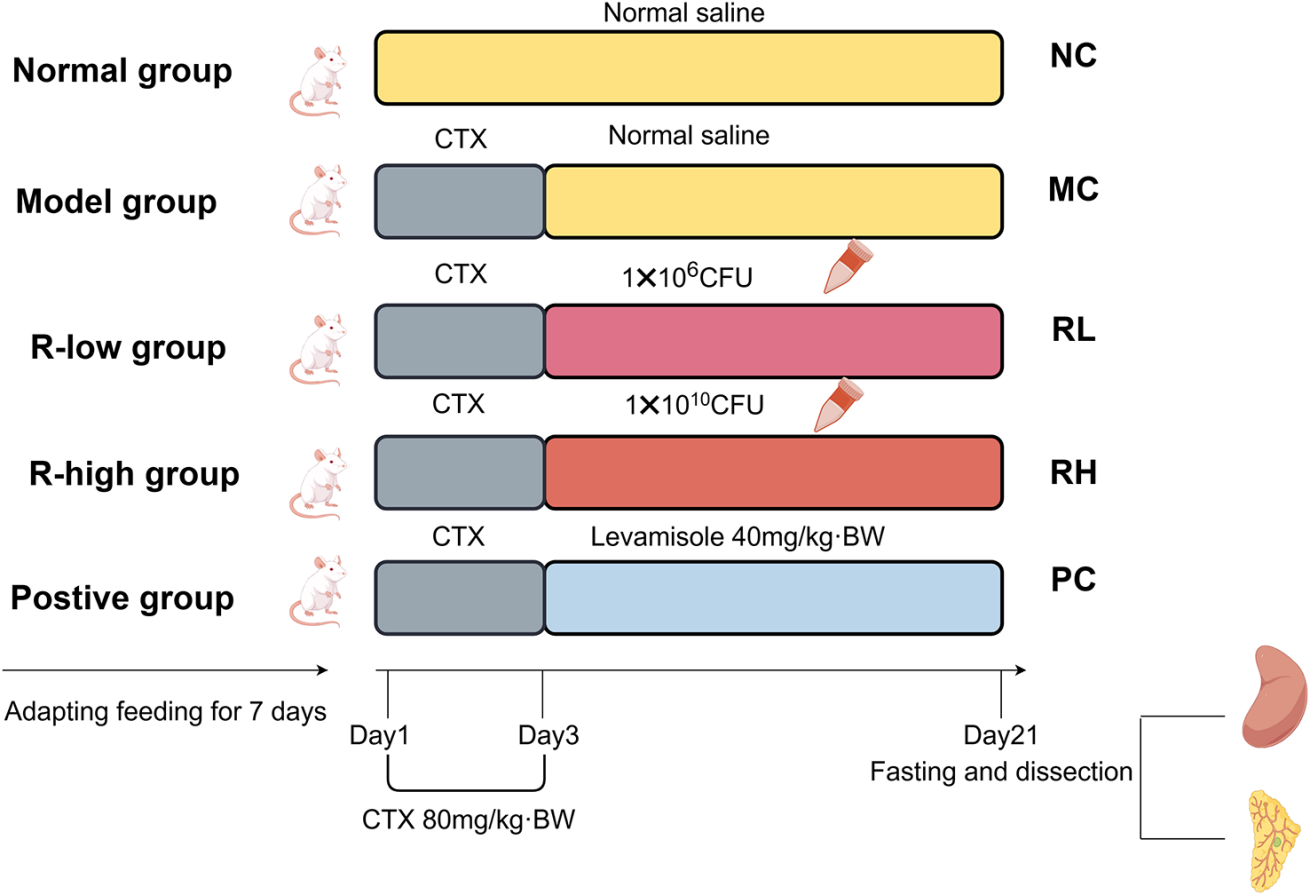
Classification, modelling and administration of mice in each group.

Sixty male SPF-grade BALB/C mice, 22 ± 2 g were purchased from Liaoning Changsheng Biotechnology Company Limited (Benxi, China). The mice were housed under controllable condition (22 ± 1°C, 12 h light/dark cycle) and had free access to water and food. After 7 days of adaptive feeding with the diet, the mice were randomly divided into 5 groups, with 12 mice in each group: normal control group (NC), model control group (MC), two experimental groups of JAASSRY1 (RL:1.0 × 10^6^ CFU/ml, RH 1.0 × 10^10^ CFU/ml, 0.5 ml/100 g/bw/day), and positive drug group (levamisole hydrochloride: 40 mg/kg, PC). From day 1 to day 3 of the experiment, except for the NC group which was intraperitoneally injected with normal saline, the other groups were intraperitoneally injected with CTX (80 mg/kg/bw) for 3 consecutive days to establish a mouse model of immunodeficiency. Starting from day 4 of the experiment, except for the NC and MC groups which were intragastrically administered with normal saline, the other groups were intragastrically administered with the corresponding doses until the end of the experiment. The specific grouping is shown in Fig. 1. This animal experiment was approved by the Animal Protection and Use Committee of Jilin Academy of Agricultural Sciences (Northeast Innovation Center of China Agricultural Science and Technology) (LAMECJAAS-2024-031).

### Determination of Body Weight and Immune Organ Index

One night before the formal start of the experiment, mice in each group were fasted for 12 h, and then the fasting body weight of each group of mice was measured and recorded as the initial body weight. The body weights of mice in each group were recorded at the end of modeling, on the fourth day of the experiment. After the last gavage, mice were fasted for 12 h that night, and the fasting body weights of mice in each group were measured the next day and recorded as the final body weight. Subsequently, the mice were sacrificed for tissue collection and weighing. The spleen index and thymus index of the mice were calculated and analyzed according to the following formulas.


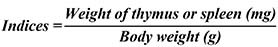


### Hematoxylin–Eosin(H&E) Staining Analysis

Fresh mice spleen tissues were collected and fixed in 4% paraformaldehyde solution overnight. The fixed spleens were subjected to experimental operations such as paraffin embedding, sectioning, dewaxing, staining, and mounting. Histological images were acquired using CaseViewer Version: 2.4. Manufacturer: Developed by 3D Histech from Hungary for histopathological evaluation of the central area.

### Determination of Splenic Immune Factor Levels (ELISA Kit Method)

Prepare a 10% tissue homogenate from the mouse spleen tissue. After centrifuging at 3,000 r/min for 15 min, collect the supernatant. Determine the protein concentration of the supernatant using the BCA method, and measure the levels of IL-4, IL-6, IL-17, IgA, IgG, and IFN-γ using an Interleukin-4 (IL-4) kit, Interleukin-6 (IL-6) kit, Interleukin-17 (IL-17) kit, Interferon-γ (IFN-γ) kit, Immunoglobulin G (IgG) kit, Immunoglobulin A (IgA) kit, Beijing Ruida Henghui Technology Development Co., Ltd., (China). During the experiment, strictly follow the instructions of the kit.

### Immunofluorescence Assay for Protein Expression in Spleen Tissues

After fixation, permeabilization, and blocking, the spleen tissues from each experimental group were incubated overnight at 4°C with Rabbit anti-TLR4, MyD88, NFκb p65, Bax, Bcl-2, mouse anti-β-actin, Wuhan Sanying Biotechnology Co., Ltd., China. The next day, the primary antibodies were washed off with PBS. Appropriately diluted fluorescent secondary antibodies were added and incubated for 1 h in the dark, followed by washing with PBS. Nuclei were stained with DAPI. Anti-fade mounting medium was used to preserve signal integrity. Histological images were captured using Case Viewer software and quantified using Image J software.

### Western Blot (WB) Analysis

The Western Blot analysis method was used to determine the expression levels of TLR4, MyD88, NF-κB p65, p-IkBa, Bcl-2, and Bax proteins in spleen tissue. β-actin was selected as the internal reference protein. PMSF lysis buffer was added to the spleen to prepare tissue homogenate, which was fully lysed on ice for 1 h. Proteins were extracted, and the protein concentration was detected according to the instructions of the BCA kit. Proteins were separated by 10% SDS-PAGE gel electroscopes and transferred to a PVDF membrane. The membrane was blocked with 5% non-fat milk for 2 h. The primary antibodies (β-actin was diluted at 1:20000, and the others were diluted at 1:2000) were incubated overnight at 4°C. The membrane was washed with TBST for 5 min, repeated 5 times. After incubating the secondary antibody at room temperature for 1 h, the membrane was washed with TBST for 6 min, repeated 5 times, and then photographed using a bioluminescence imaging system. Image J software was used for quantitative analysis, with β-actin as the internal reference.

### Gut Microbiota Analysis

After the gavage on the last day, the feces of the mice were collected and stored at -80°C for later use. The DNA of the mouse feces was extracted using a kit, and high-throughput sequencing of the 16S rRNA gene of the gut microbiota was performed to analyze the species composition of the gut microbiota and its changes at the phylum and genus levels (Shanghai Parsonage Biotechnology Co., China).

### Data Processing

All experimental data were repeated three times, and the results were expressed as mean ± standard deviation. Significance analysis was performed using SPSS Version: 25.0 Manufacturer: IBM Co. USA, with one-way analysis of variance and Duncan's test. *P* < 0.05 indicated a significant difference, and *P* < 0.01 indicated an extremely significant difference. GraphPad Prism V8 Version: 8.0.2.263. Manufacturer: GraphPad Software, LLC, (USA) was used for plotting. The Classification, modelling and administration of mice in each group was drawn on Figdraw(https://www.figdraw.com).

## Results

### Effects of JAASSRY1 on Body Weight, Organ Index, and Histopathology in Mice

**Fig. 2:**
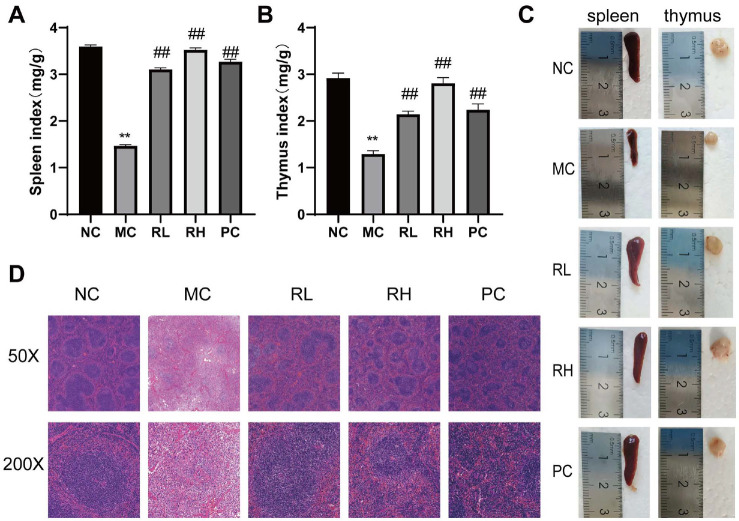
Effects of *Rhodotorula mucilaginosa* on immune organ in mice.

Table 1 shows the initial, post-modeling, and final body weights of mice in each group. At the beginning of the experiment, the body weights of each group were basically the same, and there was no significant difference among groups. After three consecutive days of intraperitoneal injection of CTX for modeling, the body weights of mice in all groups except the NC group significantly decreased (*P* < 0.01). On the last day of the experiment, compared with the NC group, the body weight of mice in the MC group decreased significantly decreased (*P* < 0.01); compared with the MC group, the body weight of mice in the RL group increased significantly (*P* < 0.05), and the body weights of mice in the RH and LH groups increased significantly decreased (*P* < 0.01). The results indicate that JAASSRY1 can alleviate the body weight loss of mice caused by CTX in a dose-dependent manner. Fig. 2A-2C shows the effects of JAASSRY1 on the immune organs of mice. Compared with the NC group, the thymus and spleen of mice in the MC group were atrophied and smaller, and both the thymus index and spleen index decreased significantly decreased (*P* < 0.01), indicating that CTX caused atrophy of immune organs and had obvious immunosuppressive effects. Compared with the MC group, the thymus and spleen indices of the RL, RH, and PC groups increased significantly (*P*<0.01). This indicates that JAASSRY1 can inhibit the atrophy of the thymus and spleen of mice caused by CTX, reduce the damage of CTX to immune organs, and show a dose-dependent relationship.

Fig. 2D illustrates H&E staining of mouse spleen tissue pathology. In the NC group, spleen tissue displayed typical morphology with a smooth surface and intact structure, clearly delineating the white and red pulp. The white pulp was stained a deep blue, indicating densely packed lymphocytes. Conversely, the MC group exhibited severe spleen damage and pathological alterations. The white and red pulp structures were disordered with blurred boundaries, lighter staining in the white pulp, reduced lymphocyte count, twisted and deformed splenic aorta, and loose splenic trabeculae. In contrast, the RL, RH, and PC groups showed alleviated spleen damage compared to the MC group. Their white and red pulp boundaries were clearer, white pulp staining was more intense, and lymphocyte numbers were higher, indicating improved overall structure. This suggests that JAASSRY1 enhances splenic lymphocyte proliferation, mitigates CTX-induced spleen damage, and boosts immune function in mice.

### Effects of JAASSRY1 on Immune Factors and Immunoglobulins in the Spleens of Mice

**Fig. 3:**
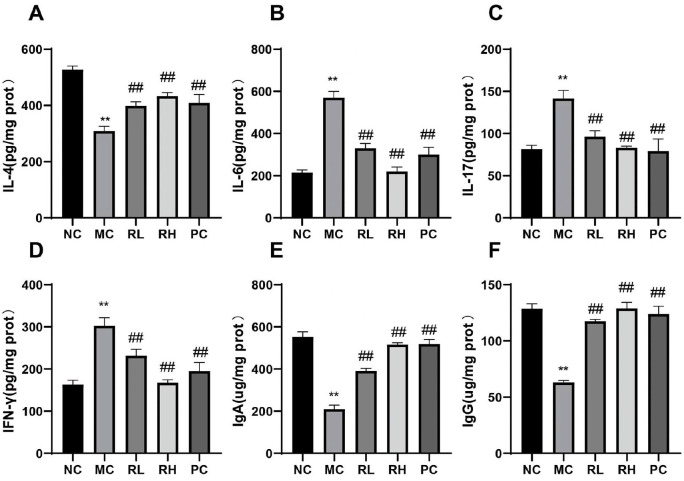
Effects of *Rhodotorula mucilaginosa* on IL-4, IL-6, IL-17, IFN-γ, lgA, lgG content in mice spleen.

Fig. 3 shows the effects of JAASSRY1 on the expression levels of pro - inflammatory cytokines IL-6, IL-17, IFN-γ, anti-inflammatory cytokine IL-4, and immunoglobulins IgA and IgG in the spleens of mice. Compared with the NC group, the levels of IL-6, IL-17, and IFN-γ in the MC group were significantly increased (*P* < 0.01), while the contents of IL-4, IgA, and IgG were significantly decreased (*P* < 0.01). When compared with the MC group, all the indicators in the RL, RH, and PC groups showed a significant reversal (*P* < 0.01). The results indicate that JAASSRY1 can effectively alleviate the immunosuppression caused by CTX in mice, reduce inflammation, and enhance the immunity of immunocompromised mice.

### Effects of JAASSRY1 on the TLR4/MyD88/NF-κB Pathway in the Spleens of Mice

**Fig. 4:**
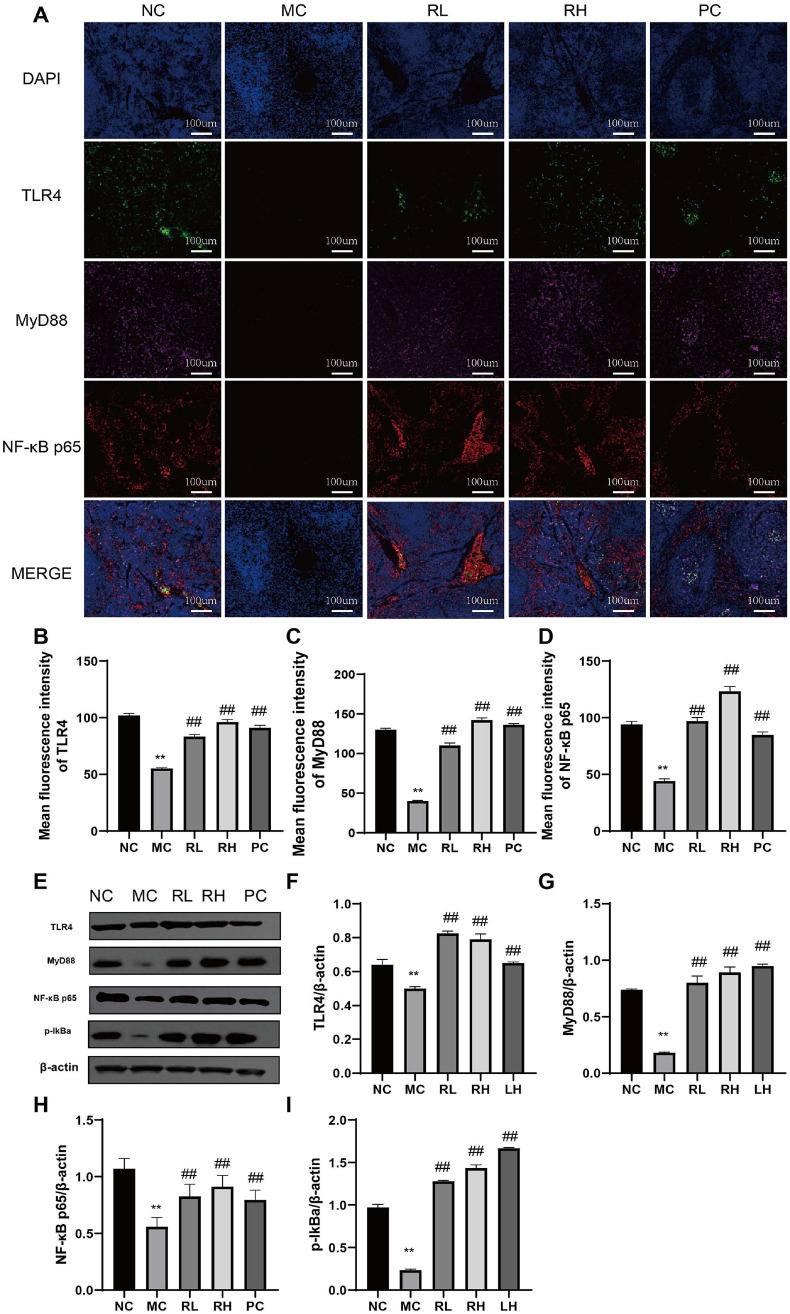
Effects of *Rhodotorula mucilaginosa* on TLR4/MyD88/NF-κb signaling pathway in spleen.

The immunofluorescence analysis results of the NF-κB pathway in the mouse spleen are shown in Fig. 4A-4D. The expression levels of TLR4, MyD88, and NF-κB p65 in the MC group were significantly lower than those in the NC group (*P* < 0.01). WB was further used to analyze the protein expression levels in the spleen, and the results were consistent with the immunofluorescence findings (Fig. 4E-4I). That is, compared with the MC group, the expression levels of TLR4, MyD88, NF-κB p65, and p-IKBα in the RL and RH groups increased significantly (*P* < 0.01). These results indicate that JAASSRY1 can upregulate the expression levels of TLR4, MyD88, NF-κB p65, and p-IKBα proteins in the mouse spleen.

### Effects of JAASSRY1 on the Bcl-2/Bax Pathway in the Spleens of Mice

**Fig. 5:**
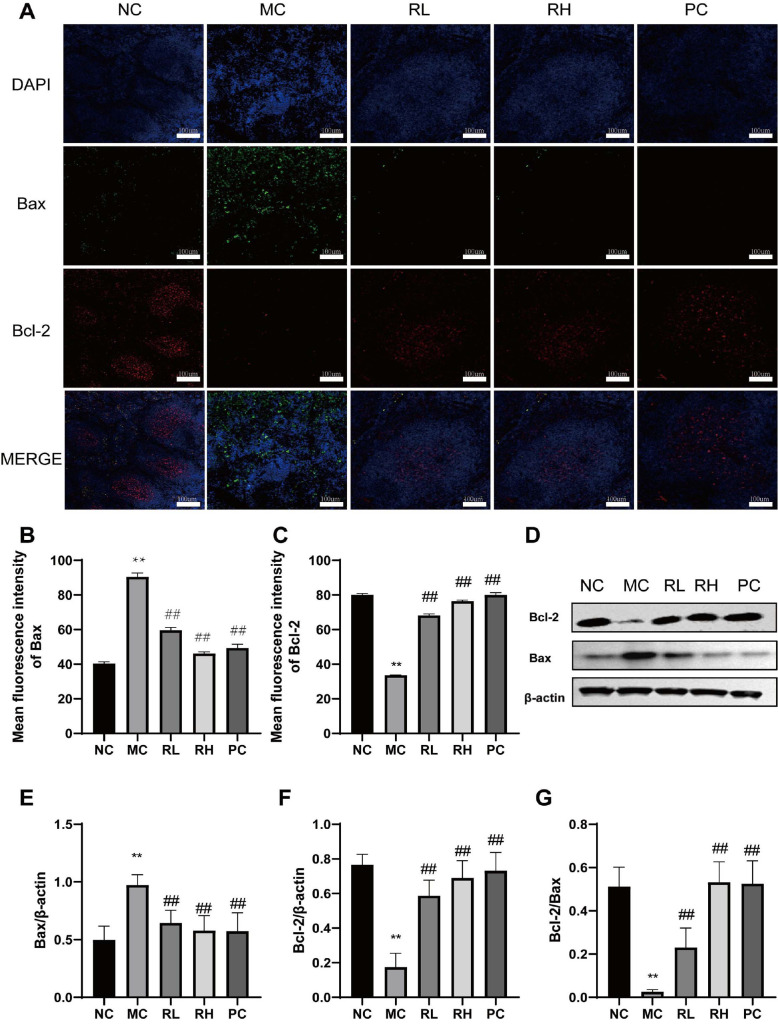
Effects of *Rhodotorula mucilaginosa* on Bax/Bcl-2 signaling pathway in spleen

The results of immunofluorescence analysis of Bcl-2 and Bax-related proteins in mouse spleens are shown in Fig. 5A-5C. The expression level of Bcl-2 in the MC group was significantly lower than that in the NC group (*P* < 0.01), while the expression level of Bax was significantly increased (*P* < 0.01), and the Bcl-2/Bax ratio was significantly decreased (*P* < 0.01). WB was used for further verification, and the trend was consistent (Fig. 5D-5G). Compared with the MC group, the expression levels of Bcl-2 in the RL and RH groups were significantly increased (*P* < 0.01), the expression levels of Bax were significantly decreased (*P* < 0.01), and the Bcl-2/Bax ratio was significantly increased (*P* < 0.01). These results indicate that JAASSRY1 can improve mouse spleen injury by regulating the expression levels of Bcl-2 and Bax proteins in mouse spleens and increasing the Bcl-2/Bax ratio.

### Effects of JAASSRY1 on the Gut Microbiota of Mice

**Fig. 6:**
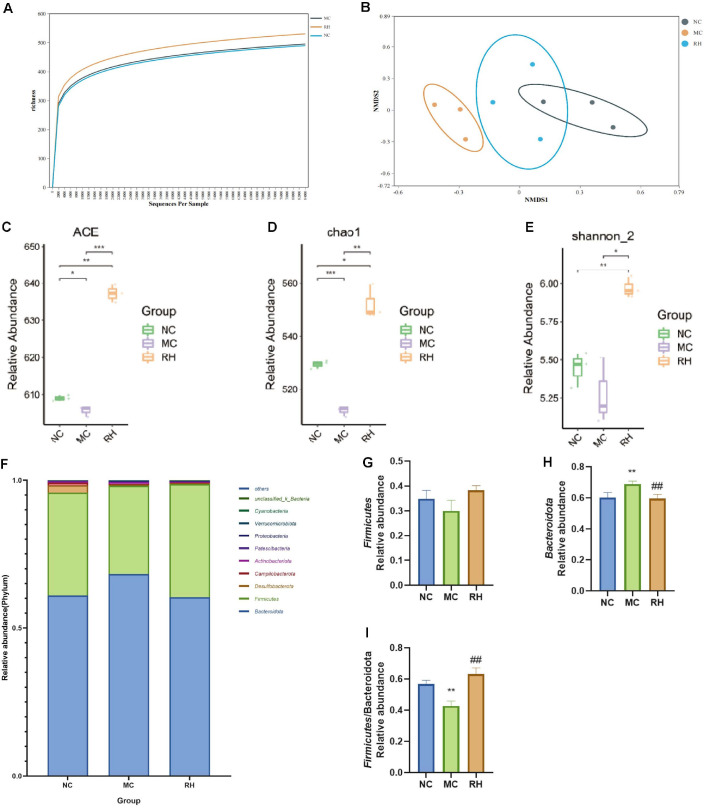
Effects of *Rhodotorula mucilaginosa* on the intestinal flora of mice.

There is a close interaction between the gut microbiota and the host immune system. The changes in the gut microbiota were further investigated by 16S rRNA sequencing. When the sequencing depth reached around 30,000, the curve flattened out, indicating that most of the species had been detected and the sequencing depth was reasonable (Fig. 6A). The non-metric multidimensionalscaling (NMDS) results showed that the NC group and the MC group could be completely separated, and the RH group was closer to the NC group, suggesting that the composition of the gut microbiota in the RH group was more similar to that in the NC group (Fig. 6B). In addition, there were also significant differences in the microbial communities between the MC group and the RH group, indicating that the community composition of the gut microbiota was affected by JAASSRY1. The α-diversity results (Fig. 6C-6E) showed that the ACE, Shannon-2, and Chao1 indices in the MC group were significantly lower than those in the NC group (*P* < 0.01), and the indices in the RH group were significantly higher than those in the MC group (*P* < 0.05), suggesting that JAASSRY1 could restore the microbial diversity in immunosuppressed mice and maintain gut homeostasis. The relative abundances of the gut microbiota in mice at the phylum level mainly included *Bacteroidota* and *Firmicutes*. CTX increased the relative abundance of *Bacteroidota* (*P* < 0.01) and decreased the relative abundance of *Firmicutes*, and the F/B ratio was significantly reduced (*P* < 0.01). The RH treatment significantly inhibited the overgrowth of *Bacteroidota*, and the F/B ratio was significantly increased (*P* < 0.01) (Fig. 6F-6I).

**Fig. 7:**
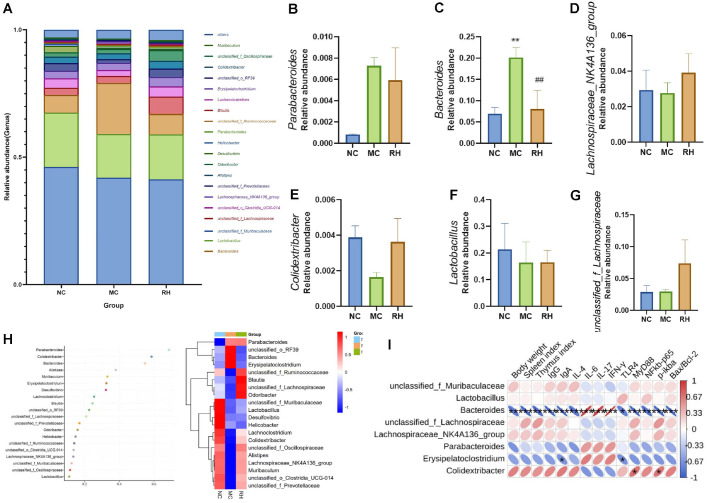
Genus level changes of intestinal flora in mice and correlation analysis.

As shown in Fig. 7A-7G, the distribution of gut microbiota at the genus level varied among different groups. Compared with the NC group, the MC group showed a decrease in the abundances of *Lachnospiraceae*_NK4A136_group, *Colidextribacter*, and *Lactobacillus*, an increase in the abundance of *Parabacteroides*, and a significant increase in the abundance of *Bacteroides* (*P* < 0.01). RH effectively reversed these changes, and the relative abundance levels of *Bacteroides* and *Colidextribacter* returned to normal, with the change in the relative abundance of *Bacteroides* being statistically significant (*P* < 0.01). Combining with the random forest plot (Fig. 7H), it was found that there were significant differences in gut microbiota between the MC group and the NC group, and the RH group reversed these changes. *Parabacteroides*, *Colidextribacter*, and *Bacteroides* were the marker species for the inter-group differences and could serve as characteristic bacteria at the species level of gut mucosal microbiota. Therefore, JAASSRY1 can improve CTX-induced gut microbiota changes by regulating the levels of bacterial phyla and genera, especially the relative abundance of *Bacteroides*.

To identify the gut microbiota that may contribute to the regulation of immune function in immunosuppressed mice, Pearson correlation analysis was conducted between the top 8 genera in terms of abundance at the genus level, immune-related indicators, and spleen-related pathway proteins. The results are shown in Fig. 7I. *Bacteroides* was significantly positively correlated with IL-6, IL-17, and IFN-γ (*P* < 0.01), and significantly negatively correlated with the relative expression levels of proteins related to mouse body weight, thymus index, spleen index, IL-4, IgA, IgG, TLR4, MyD88, NF-κB, and Bax/Bcl-2 (*P* < 0.01). *Colidextribacter* was positively correlated with the expression levels of MyD88 and p-IKBa (*P* < 0.05). Therefore, the changes in gut microbiota after JAASSRY1 intervention are closely related to the improvement of host spleen immune performance and inflammation. In particular, the abundance of *Bacteroides* is negatively correlated with immune performance.

## Discussion

Immunosuppression is a state of long - term or temporary immune system dysfunction. Since many diseases are associated with immune system dysregulation, immunomodulation plays a crucial role in maintaining body health. CTX as an immunosuppressant, is commonly used in cancer treatment. However, it can inhibit the body's immune system by increasing the spleen index, reducing the thymus index, damaging the intestinal mucosal barrier, and causing dysregulation of the composition and structure of gut microbiota [18, 19]. This study showed that after three consecutive days of CTX induction, the body weight of mice decreased significantly and grew slowly thereafter. Related studies have indicated that body weight decreases significantly after CTX injection and remains low for a long time, which is also confirmed by our data [20]. The spleen and thymus are two important lymphoid organs for the differentiation and maturation of immune cells. Their functional states are closely related to the host's immune function, and their organ indices are indicators for evaluating the overall immune state of the body [21]. In this study, the spleen and thymus indices of the MC group were significantly decreased lower than those of the NC group (*P* < 0.01). After JAASRY1 intervention, the spleen and thymus indices of the RL and RH groups were higher than those of the MC group, and the differences were significant, which is consistent with the conclusion of Ge *et al*. [12] and contradicts the research results of Shen *et al*. [22] The different trends of immune organ indices in the CTX group suggest that CTX causes different degrees of immune damage in different animal species. Pathological analysis showed that in the MC group, the structure of the lymphatic sheaths around the splenic lymph was blurred, splenocytes were ruptured, the boundary between the red pulp and white pulp was unclear, and the splenic trabeculae were broken. After the action of JAASRY1, the structure of the splenic trabeculae in mice was normal, and the boundary between the red pulp and white pulp was clear. This indicates that JAASRY1 can increase the immune organ indices of mice and restore the normal morphology of the spleen.

IL-4 plays roles in promoting the differentiation of Th0 cells and stimulating the proliferation of activated B cells and T cells in the body's immune response [14]. IL-6, a cytokine secreted by macrophages, can promote B cell proliferation and positively regulate natural killer (NK) cells [23]. IL-17, mainly secreted by Th17 cells, maintains a pro - inflammatory environment and may cause tissue damage [24]. IFN-γ, primarily secreted by activated T lymphocytes, serves as an important immunomodulatory cytokine in the body, with functions such as activating Th1 cells and mediating cellular immunity [25]. The level of serum immunoglobulin (Ig) reflects humoral immune function [26]. This study found that compared with the NC group, JAASRY1 could significantly increase the levels of IL-4, IgA, and IgG in the spleen and reduce the contents of IFN-γ, IL-6, and IL-17. This indicates that JAASRY1 can activate lymphocytes to produce antibodies or cytokines, thereby regulating cellular immune responses and humoral immune functions and restoring the secretion of immune cytokines and immunoglobulins to normal levels. The reason for this is that *R. mucilaginosa* is rich in carotenoids, whose structural characteristics endow it with strong antioxidant capacity, and the polysaccharides in its cell wall have the ability to activate the immune system [14, 15]. For example, carotenoids can reduce the levels of pro - inflammatory cytokines IFN-γ and IL-6 [27], promoting immune regulation. However, Li Xueqiang *et al*. found that yeast culture increased the contents of IL-1β, IL-6, and IFN-γ in the serum of Simmental cattle [28]. This difference in results may stem from the changes in the active components of *R. mucilaginosa* caused by the use of different strains, which requires in - depth exploration.

To further elucidate the protective mechanism of JAASRY1 against CTX-induced immunosuppression, the expression levels of the TLR4, MyD88, and NF-κB p65 signaling pathways were systematically evaluated. As a member of the TLR family, TLR4 can be activated by lipopolysaccharide [29]. MyD88, a downstream adaptor molecule of TLR4, is immediately activated after the activation of cell-surface TLR4. Subsequently, the activated MyD88 phosphorylates the IκBa complex, thereby activating NF-κB [22]. This study found that CTX treatment significantly reduced the expression levels of TLR4, MyD88, and NF-κB p65 proteins, which is consistent with previous research findings [30-32]. Di Tomo *et al*. [33] confirmed that β-carotene can promote TNF-α induced NF-κB activation, exerting anti-inflammatory and immunomodulatory properties, which is consistent with our experimental results. Relevant studies have also shown that the synergistic anti-inflammatory effects of lycopene, lutein, β-carotene, and carnosic acid are achieved by inhibiting NF-κB signaling [30]. These seemingly contradictory results emphasize the dual regulatory role under different immunomodulatory conditions, which may confer unique therapeutic effects in different clinical settings. Baek *et al*. [34]reported that yeast cell wall polysaccharides can activate the MAPK/NF-κB pathway. These findings suggest that the regulation of the splenic TLR4/MyD88/NF-κB pathway by JAASRY1 may be regulated by its active components, carotenoids, and cell wall polysaccharides.

The Bcl-2/Bax pathway is a key regulator of apoptosis and cell survival and is an integral part of CTX-induced spleen injury [34]. The Bcl-2 family consists of proteins with opposing effects: anti-apoptotic and pro-apoptotic [36]. Bax, a pro-apoptotic protein, promotes apoptosis. Maintaining an appropriate balance between Bcl-2 and Bax is crucial for regulating mitochondrial apoptosis. A study by Xie *et al*. [37] showed that G-EMP (Exopolysaccharides from Genistein) improved CTX-induced intestinal injury by downregulating the ratio of apoptotic proteins (Bax/Bcl-2). Özge *et al*. [38]found that hesperidin (HES) effectively alleviated isfosfamude (IFA)-induced nephrotoxicity by inhibiting the level of Bax and increasing the level of Bcl-2. These results are consistent with the present study, in which JAASSRY1 significantly regulated the ratio of apoptotic proteins (Bax/Bcl-2) to mitigate CTX-induced immunosuppression.

The gut microbiota is a collective term for the trillions of bacteria in the gut, playing a fundamental role in regulating host physiology and immunity. CTX typically leads to gut ecological disorder, reduces microbial community richness, promotes the growth of harmful bacteria, and inhibits the growth of beneficial bacteria [39]. High diversity of the gut microbiota is beneficial for maintaining gut stability. The α-diversity analysis of gut species can reflect the richness and diversity of the microbial community, and the reduction in richness and diversity is often associated with abnormal immune function [40]. This study found that CTX could cause a decline in the ACE, Chao 1, and Shannon indices of the gut microbiota in mice, similar to the findings of Cui *et al*. [41], indicating that CTX reduces the diversity and richness of the gut microbiota, while JAASSRY1 reverses these changes. Our study shows that *Bacteroidota* and *Firmicutes* dominate at the phylum level in mice. After CTX modeling, the relative abundance of *Bacteroidota* significantly increased, and the *Firmicutes*/*Bacteroidota* (F/B) ratio significantly decreased. *Bacteroidota* is one of the most abundant Gram-negative anaerobic bacteria in the human gut, participating in various metabolic diseases and unique bacteria related to oxidative stress in the human body. Its abundance is positively correlated with inflammatory markers, promotes inflammation, and plays a crucial role in regulating the human immune system. Moreover, an imbalance in the F/B ratio may lead to immune dysfunction [24, 40]. With the intake of JAASSRY1, the relative abundance of *Bacteroidota* significantly decreased (*P* < 0.01), and the F/B ratio significantly increased (*P* < 0.01), thereby improving dysbiosis. At the genus level, CTX treatment was found to increase the relative abundance of *Parabacteroides* and *Bacteroides*, decrease the relative abundance of *Colidextribacter*. This easily induces ecological imbalance of the gut microbiota and affects the symbiotic relationship between the gut microbiota and the host, thereby affecting immune system function. *Parabacteroides* plays a dual role in many human diseases. The abundance of *Parabacteroides* increases in patients with hypertension, polycystic ovary syndrome, and non-alcoholic steatohepatitis (NASH) [15, 42]. Our study found that with the intake of JAASSRY1, the relative abundance of *Parabacteroides* decreased, and immunosuppression was improved. However, interestingly, in human studies, *Parabacteroides* was found to play an anti-inflammatory role by alleviating the release of IL-8 from LPS-induced HT-29 cells [43], and the host benefits from the presence of *Parabacteroides*. This indicates that the pathological role of *Parabacteroides* in diseases may depend on the environment, including the host's susceptibility to immunosuppression, impaired bacterial clearance, and promotion of a high inflammatory response, coupled with differences between strains, which still require further research in the future. Little is known about the function of *Colidextribacter* in metabolic pathways related to human health, and there are contradictions in the literature regarding the harmful or beneficial effects of *Colidextribacter*. In an experimental model of liver fibrosis, this bacterium was identified as a harmful species that damages the gut barrier function [44]. Conversely, a study on hyperuricemia rats found that *Colidextribacter* is a beneficial bacterium [45], supporting our conclusion. *Bacteroides* has been proven to be able to regulate the functions of immune cells, especially dendritic cells and macrophages, thereby promoting an anti-inflammatory phenotype [43]. The imbalance of *Bacteroides* is associated with various health conditions, including inflammatory bowel disease (IBD) and metabolic disorders. Its role in maintaining gut homeostasis makes it the focus of potential therapeutic interventions [46]. This study found that CTX led to a significant increase in the abundance of *Bacteroides* (*P* < 0.01), indicating that the abnormal increase in *Bacteroides* abundance disrupts the overall balance of the gut microbiota, resulting in poor health conditions [46]. Meanwhile, correlation analysis showed that the relative abundance of *Bacteroides* was significantly negatively correlated with immune-related indicators such as mouse body weight, thymus index, and spleen index (*P* < 0.01), negatively correlated with the relative expression levels of spleen proteins TLR4, MyD88, NF-κB p65, and Bax/Bcl-2, and negatively correlated with immunoglobulins IgA and IgG (*P* < 0.01); it was strongly positively correlated with inflammatory expression (*P* < 0.01). That is, the abnormal increase in *Bacteroides* abundance is related to gut microbiota disorder, inflammation, and impaired spleen immune function, while the supplementation of JAASSRY1 reverses these changes. In this study, after JAASSRY1 intervention, the relative abundance of *Parabacteroides* and *Bacteroides* decreased, and the relative abundance of *Colidextribacter* increased, regulating the balance of the gut microbiota. Among them, the abundance of *Bacteroides* was adjusted to the normal state, making it a potential core microbiota for restoring gut homeostasis and promoting immune function recovery.

## Conclusion

This study investigated the immunomodulatory effects of *R. mucilaginosa* JAASSRY1 in a CTX-induced immunosuppression model. Intervention with *R. mucilaginosa* JAASSRY1 could alleviate weight loss and immune organ atrophy, restore the normal secretion of immune cells and immunoglobulins, and enhance immune function. Meanwhile, it could target and regulate the TLR4/MyD88/NF-κB pathway and apoptosis-related proteins (Bax/Bcl-2) in the mice spleen, thereby restoring the immune performance of the spleen. Additionally, it could modulate the composition of gut microbiota and restore gut homeostasis. Correlation analysis indicated that the changes in gut microbiota were strongly correlated with the alterations in immune-related indicators, and they could act synergistically to reconstruct the immune function of immunosuppressed mice. In conclusion, *R. mucilaginosa* JAASSRY1 can improve immunosuppression through the synergistic effect of the “gut microbiota-spleen” axis, providing new insights for its subsequent development and utilization.

## Figures and Tables

**Table 1:** Effects of *Rhodotorula mucilaginosa* on body weight in mice (g).

Group	Body mass/g		
	1 d	4 d	24 d
NC	23.27 ± 0.80	24.71 ± 0.59	27.68 ± 0.89
MC	23.03 ± 0.57	20.87 ± 0.73**	24.09 ± 0.53**
RL	23.16 ± 0.65	20.95 ± 0.84**	25.66 ± 0.43^#^
RH	23.26 ± 0.35	20.58 ± 0.52**	26.57 ± 0.46^##^
PC	23.13 ± 0.82	20.99 ± 0.63**	26.38 ± 0.59^##^

Data are presented as mean ± SD. *P < 0.05, ** *P* < 0.01 represent significant differences between the NC and other groups ; #*P* < 0.05, ##*P* < 0.01 represent significant differences between the MC and other groups.
